# Functional Diagnosis in Albuminuria

**Published:** 1919-07-26

**Authors:** 


### DISEASE AND THE LABORATORY.

### I. Functional Diagnosis in Albuminuria.

Of the various organs in the body the kidneys occupy an exceptionally favourable position for quantitative estimation of their functional activity. The methods available are either to compare the concentration of a substance normally present in the blood plasma with its rate of excretion in the urine, or to inject into the body a known dose of a foreign substance and then determine its rate of excretion. In the former method urea and chloride are the substances studied; in the latter method, various substances have been tried, such as sodium-sulphindigotate and methylene blue, which have proved to be unsatisfactory and have been replaced by the sodium salt of phenol sulphone phthalein, sometimes known as phenol red.

### Urea Determinations.

Systematic determinations of urea in blood and blood-serum were first performed by Widal and his pupils in Paris. They found that the results obtained for blood differed inappreciably from those for serum, and that the results were consistent and gave a valuable index of the functional activity of the kidneys and of the possibility of the onset of uræmia. Why previous results had been confusing is not clear, but the methods employed may have had something to do with it.

The normal value varied from two to five parts of urea in 10,000 of blood, and on an ordinary diet was not above three parts per 10,000. By no means all renal cases showed higher urea in the blood than the normal, but those that did, belonged to definite types, while in uræmia there were very high figures, thirty per 10,000 or higher. Typical cases of contracted kidney with dilute urine, high blood-pressure, hypertrophied heart and commencing albuminuric retinitis often gave figures as high as ten per 10,000, even long before death actually took place. These cases showed the effect of diet very markedly, giving a higher figure on a diet rich in nitrogen, while on a low-nitrogen diet there was a marked fall, even to a figure within normal limits. To obviate this difficulty Ambard determined the laws governing the relation of the rate of urea excretion to the concentration in the blood, and devised a method of calculating, from simultaneous determinations of urea in blood and in urine, a figure known as Ambard's constant, which varies in the normal from 0.068 to 0.080 and is independent of the diet and not affected by diuretics. As with the urea in blood, this figure rises with renal impairment.

The French workers determined urea by the hypobromite method. With blood, of which 10 ccm. is sufficient, the proteins were removed at first by adding alcohol, filtering and evaporating at low temperature, but afterwards trichlor-acetic acid was found more convenient for precipitating the protein, as no evaporation was then needed. For the actual determination the apparatus of Yvon was used; this has the advantage, where small quantities of urea are concerned, that there is no air in the apparatus when the reaction starts. The results including the values of Ambard's constant have since been confirmed in America by McLean using the urease methods, and he has also introduced into the formula a correction for the weight of the individual, which is obviously necessary, though he probably over-corrects, as he assumes the renal value to be directly proportional to the weight of the individual, not to his surface.

### Chloride Determination.

Determinations of chloride in blood plasma and urine have been dealt with in a similar way. Here serum or plasma must be used, as the chloride of the red corpuscles is essentially different from that of the plasma. The relation between plasma and urine differs in the case of chloride from that in the case of urea; for chloride there is a threshold value (5.62 parts of sodium chloride per 1,000 of plasma) below which chloride is not excreted by the kidneys, and the rate of excretion is dependent on the amount by which the chloride of the plasma exceeds this value. The results show that, where there is retention of urea, there is also retention of chloride, but the converse does not hold, as there is a group of cases of nephritis with œdema which show no retention of urea, but show marked retention of chloride with high figures for the plasma and low figures for the urine.

### Phenol Sulphone Phthalein Test.

In the phenol sulphone phthalein test of Rowntree and Geraghty the method is as follows:—The bladder is first emptied by catheter if there is any residual urine; a quantity of the sodium salt of the dye is then injected intramuscularly, preferably into the lumbar muscles, and the urine is voided two hours after the time of the injection. Sufficient caustic soda solution is added to bring out the red colour, and water is added to make the volume up to 1,000 c.c. or other convenient known volume. The determination is then made by comparing the intensity of colour with that of a solution containing 6 milligrammes of the dye per 1,000 c.c., together with sufficient caustic soda solution, and a suitably coloured urine free from the dye is added in order to obtain a better colour match, but, of course, included in the 1,000 c.c. Glass ampoules can be obtained containing in 2 c.c. of sterile liquid 12 mg. of the dye, which is sufficient for the injection and for the standard, if less than 1,000 c.c. is made up. If no colorimeter is available, the estimation can be easily done with Nessler glasses, or with equal test tubes, diluting the standard quantitatively until a match of colour is obtained and so expressing the strength of the excreted dye in terms of the standard. The healthy kidney excretes more than 60 per cent. of that injected in the two hours, so that less than 6 per cent. means definite renal impairment.

**Disease and the Laboratory—(*continued*).**

McLean has compared the results of Ambard's constant and this method, and finds absolute agreement between the two, except that the Ambard figures fall rather more steeply as renal efficiency diminishes; thus, two cases gave respectively, Ambard figures, ratio 49.5 per cent.; and phenol red figures, ratio 63 per cent.

### Application of the Methods.

Determination of urea in blood alone is of special value with reference to uræmia, as this depends on the extent to which retention of excretory products has taken place. In typical cases the urea figure is 30 per 10,000 or higher, but in acute nephritis symptoms of uræmia may show themselves with much less retention than this. On the question of urea as a cause of the symptoms of uræmia, urea if injected in large enough doses causes death in animals with urea figures for the blood not much higher than have been found in uræmia; but death takes place in coma without convulsions and without hypernpœa.

Data have been accumulated as to the limit value for urea at which operations may be performed. This is apparently about 10 per 10,000, but doubtless depends on the nature of the operation and the anæsthetic employed.

The Ambard constant and the phenol red tests give a quantitative expression for the working kidney, and have therefore a general utility. They are specially useful in watching the progress of cases with retention, such as contracted kidney, cystic kidney, lardaceous kidney; and, further, in surgical cases the extent to which the kidneys are damaged can be estimated, which is of special value where there is a question of only one kidney being diseased, as, if the other kidney is normal, the compensating hypertrophy is usually sufficient to show practically no impairment of excretory power.

The value is also great in distinguishing those cases of albuminuria in which there is retention, such as contracted kidney, from those in which there is none or practically none, such as arteriosclerosis. As to indications for treatment, if there is nitrogenous retention, a diet poor in nitrogen is indicated, though this should apparently only be given for a limited time. High chloride in the plasma with œdema, but no nitrogenous retention, is an indication for a diet containing little chloride, as it is chloride alone that is being retained. Epstein, however, has recently suggested another form of treatment for œdematous cases. He found that the protein in the urine was mainly albumen, while the blood serum was poor in proteins, showed lipæmia and contained a high proportion of globulin to albumen. He supposed this change was due to loss of albumen in the urine, and accordingly gave a diet rich in protein and poor in fat. Some cases responded well to this treatment in that their condition was improved, the œdema disappeared, and the blood protein became more normal, though the albuminuria persisted to a certain extent.

